# Identification of novel antiviral of fungus-derived brefeldin A against dengue viruses

**DOI:** 10.1186/s41182-017-0072-7

**Published:** 2017-10-26

**Authors:** Muhareva Raekiansyah, Mihoko Mori, Kenichi Nonaka, Masanobu Agoh, Kazuro Shiomi, Atsuko Matsumoto, Kouichi Morita

**Affiliations:** 1 Department of Virology, Institute of Tropical Medicine, Nagasaki University, 1-12-4 Sakamoto, Nagasaki, 852-8523 Japan; 2 Kitasato Institute for Life Sciences, Kitasato University, 5-9-1 Shirokane, Minato-ku, Tokyo, 108-8641 Japan; 3 Graduate School of Infection Control Sciences, Kitasato University, 5-9-1 Shirokane, Minato-ku, Tokyo, 108-8641 Japan

**Keywords:** Dengue virus, Antiviral, Brefeldin A, Secondary metabolite, Fungus

## Abstract

Microbial natural products possess a wide range of biological and biochemical potential. Among them, fungal secondary metabolites are one of the most important sources for discovering new drugs or lead compounds. In the present study, we explored substances produced by the strain *Penicillium* sp. FKI-7127 for its antiviral activity. We identified brefeldin A as a novel antiviral agent against dengue viruses. The inhibitory effect of brefeldin A was confirmed by virus titer and immunofluorescence assay. Brefeldin A inhibited dengue viruses regardless of serotypes and other related viruses including Zika virus and Japanese encephalitis virus. Time-of-addition study showed that brefeldin A exerts its antiviral effect at an early stage of the dengue virus (DENV) life cycle. These studies demonstrate that (i) brefeldin A could be used as a lead compound for drug development of anti-DENV and other related viruses and (ii) fungal metabolites are a potential and valuable source for dengue virus drug discovery.

## Introduction

Dengue virus (DENV) is an important mosquito-borne pathogen for causing dengue fever (DF) and dengue hemorrhagic fever (DHF). DF is relatively mild, but DHF leads to the life-threatening dengue shock syndrome [1]. It is estimated that there are 390 million dengue infections per year, of which 96 million manifest apparently [2]. At present, no specific antiviral therapy for treatment of dengue disease is available. Thus, drug discovery research for dengue is of importance.

Natural products are valuable materials for the discovery and development of new drugs for treating many diseases since they possess a wide range of structural and functional diversity [3]. Among varied sources of natural products, secondary metabolites produced by fungi have been recognized as an important source of lead structures for new drugs [4].

Some bioactive compounds that are isolated and characterized from metabolites of soil-borne and endophytic fungi have led to the development of drugs such as anticancer drug Taxol which originated from endophytic-fungal metabolites [5]. Furthermore, a lot of antibacterial substances have been demonstrated from extracts and pure substances obtained from culture broth or fungal biomass [6]. Some studies have identified substances that inhibit viruses [7, 8]. However, there are only few reports about DENV compounds found from fungal metabolites so far [9].

In this study, we examined anti-DENV activity of secondary metabolites produced by a fungal strain, *Penicillium* sp. FKI-7127*.* We isolated and identified brefeldin A (BFA) as a novel antiviral agent against DENVs. Inhibition of BFA on Japanese encephalitis virus (JEV) and Zika virus (ZIKV) was also demonstrated.

## Materials and methods

### Cell lines and viruses

Vero cells were maintained in a minimum essential medium supplemented with 10% fetal calf serum. The cells were grown at 37 °C with 5% CO_2_. The strains used for this study were patient-derived DENV1–4 from the Philippines, strains 99st, 00st-22A, SLMC50, and SLMC318, respectively; Zika virus strain 976; and Japanese encephalitis virus (JEV) Beijing strain.

### Microorganisms and culture of the fungal strain

The fungal strain FKI-7127 was isolated from a soil around the root of *Angelica keiskei* collected in Kouzu Island, Tokyo, Japan. To observe the morphological characteristics, this strain was incubated on Miura’s medium (LcA). From the results of morphological observation, the producing strain FKI-7127 was classified as genus *Penicillium*. The strain *Penicillium* sp. FKI-7127 was maintained on an LcA slant. A loopful of spores of this strain was inoculated into a test tube, containing 10 ml of a seed medium consisting of 2% glucose, 0.5% Polypepton, 0.2% yeast extract, 0.2% KH_2_PO_4_, 0.05% MgSO_4_·7H_2_O, and 0.1% agar, and incubated for 3 days. One milliliter of the seed culture was inoculated into each of two 500-ml Erlenmeyer flasks containing 100 ml of a production medium consisting of 3% soluble starch, 1.0% glycerol, 2% soybean meal, 0.3% dry yeast, 0.3% KCl, 0.2% CaCO_3_, 0.05% KH_2_PO_4_, 0.05% MgSO_4_·7H_2_O, and 0.03% quercetin dihydrate, and the production culture was incubated for 6 days. Fifty percent of ethanol extract of cultured broth were prepared for antiviral test.

### Antigen detection ELISA

To evaluate the antiviral activity of a sample, Vero cells were seeded in 96-well plates (1 × 10^4^ cells/well) and infected with DENV at multiplicity of infection (MOI) of 0.5 in the presence of samples/compound or 0.1% dimethyl sulfoxide (DMSO). The cells were incubated for 3 days when infected culture fluid (ICF) was harvested and subjected for antigen detection enzyme-linked immunosorbent assay (ELISA) to determine dengue virus antigen level as described previously [10]. The result was expressed as a percent of inhibition which determined as (OD value of DMSO-treated cells) − (OD value of compound-treated cells) × 100% divided by (OD value of DMSO-treated cells).

### Cells viability assay

Vero cells in 96-well plates were treated with samples for 5 days. Cell viability was evaluated by MTT [3-(4,5-dimethylthiazol-2-yl)-2,5-diphenyl tetrazolium bromide] according to manufacturer’s instruction (Promega).

### Isolation and identification of brefeldin A

Cultured broth was extracted with 200 ml of ethyl alcohol (EtOH). After the mycelia were separated by centrifugation, the extract was evaporated to remove EtOH. A part of the aqueous residue was applied to a Seppak plus ODS C18 cartridge and eluted with H_2_O–CH_3_CN system to give five fractions (pass through 100:0, 70:30, 40:60, and 0:100 each 3 ml). Brefeldin A was detected in both 40:60 fraction and 0:100 fraction by high-performance liquid chromatography (HPLC) analysis, and identification of brefeldin A was achieved by high-resolution electrospray ionization mass spectrometry (HR-ESI-MS) and nuclear magnetic resonance (NMR) measurement.

### Immunofluorescence assay

Vero cells in a 24-well plate were infected with DENV-2 and added with BFA. After 48 h, infected cells were recovered, washed with PBS, and spotted onto a glass slide. Immunostaining was done as described previously [11].

### Time-of-addition studies

Time-of-addition studies were performed in 96-well plate cells as follows. (i) Pre-infection assay: Vero cells were treated with 125 nM BFA or 0.1% DMSO as control for 2 h at 37 °C prior to being washed twice with PBS and infected with DENV-2 at an MOI of 10. After 1.5 h virus adsorption, the cells were washed twice with PBS and incubated in fresh media for 24 h before ICFs were harvested for virus quantification. (ii) During-infection assay: Vero cells were infected with DENV-2 in the presence of BFA. After 1.5 h virus adsorption, inoculum was removed and the cells were washed twice. The cells were then incubated with fresh media for 24 h before ICFs were harvested for virus quantification. (iii) After-infection assay: Vero cells were infected with DENV-2. After virus adsorption and washing, BFA or 0.1% DMSO as control was added at seven different time points postinfection (0, 2, 4, 6, 8, 12, and 18 h). The ICFs were harvested at 24 hpi for virus quantification.

### Virus titration and focus reduction assay

Virus titers were determined using Vero cells in 96-well plates as described previously [11]. In brief, virus stock or ICFs were diluted tenfold in the MEM and inoculated to the cells. After 60 min of virus adsorption, the MEM containing 2% FCS and 1.25% methylcellulose was overlaid on the cells. The cells were then incubated for 2 to 4 days before subjected to focus staining. For focus staining, 12D11/7E8 monoclonal antibody and HRP-conjugated goat anti-mouse IgG + M were used as a primary and secondary antibody, respectively. The infected cells were visualized with 3,3′-diaminobenzidine, tetrahydrochloride (DAB). For focus reduction assay, Vero cells in 96-well plates were infected with DENV-1, 2, 3, 4, ZIKV, or JEV at an MOI of 0.5 in the presence of BFA at different concentrations. After 48 h incubation, ICFs were harvested. A hundred microliters of diluted ICFs (100× or 1000× dilution) was infected into fresh Vero cells in 96-well plates and incubate for another 2 to 4 days. The cells were then subjected for focus staining as described above. Experiments were performed twice, duplicating each.

## Results and discussion

Identification of antiviral substance in the present study was part of our drug screening study for new dengue drug discovery. We employed cell-based assay in combination with in-house antigen detection ELISA to evaluate anti-DENV activity. This technique was relatively quick and can be applied for high-throughput screening.

**Fig. 1:**
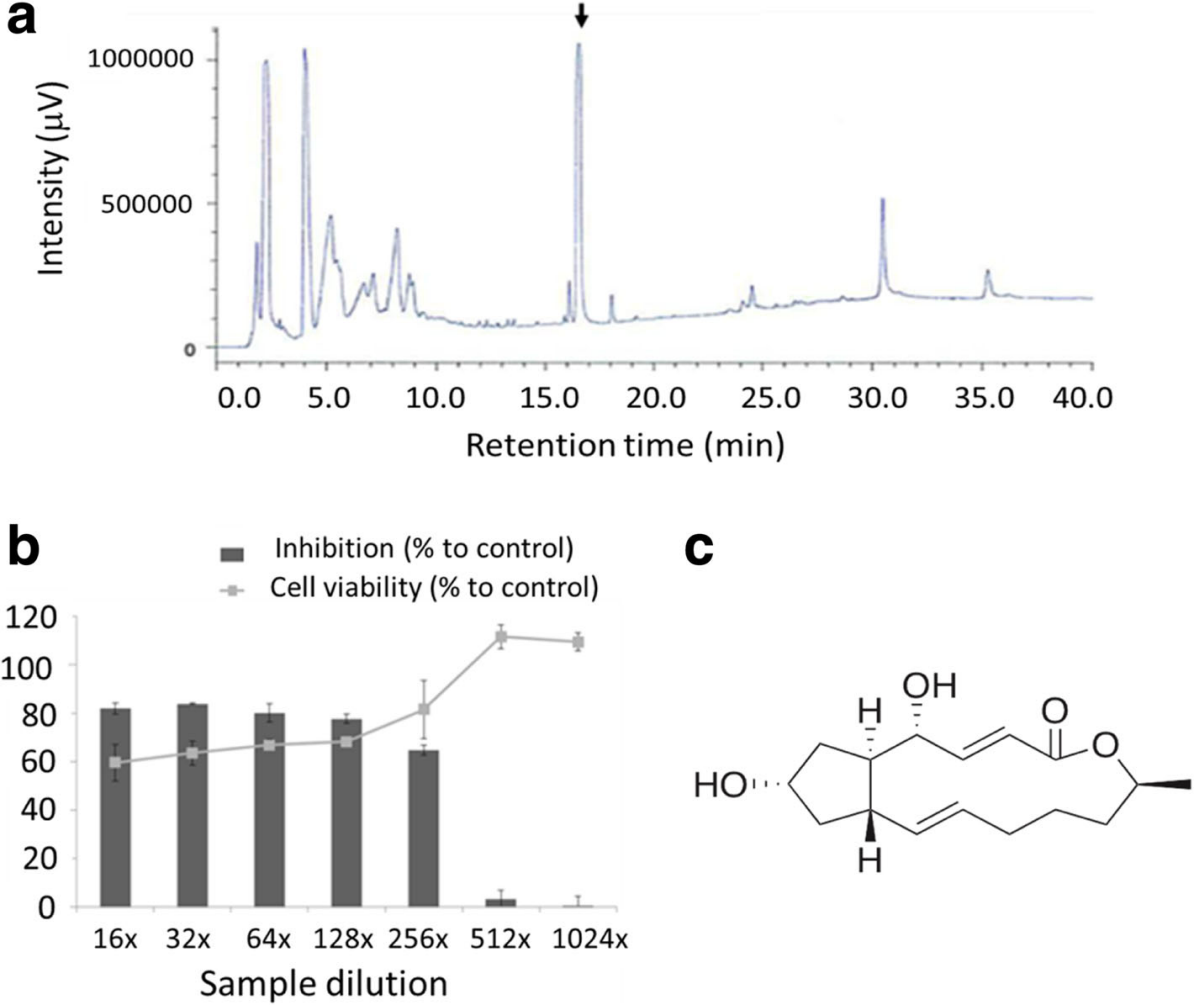
Identification of brefeldin A as DENV inhibitor. **a** Fractions of aqueous residue of cultured broth were applied to HPLC. HPLC chromatogram is shown, whereas peak of active molecule (fraction #17) was indicated with an arrow. **b** Vero cells in 96-well plates were infected with DENV at an MOI of 0.03 in the presence of fraction #17 with increasing dilutions. The cells were incubated for 5 days at 37 °C in 5% CO_2_ after which ICFs were harvested and subjected for antigen detection ELISA. In separated experiments, Vero cells in 96-well plates were treated with the fractions with increasing dilutions without virus infection. After 5 days incubation, cell viability was determined by MTT assay as described in the “Materials and methods” section. The error bars represent standard deviation from the means of the results from triplicate determination. **c** By HR-ESI-MS and NMR methods, the fraction #17 was identified as brefeldin A. Structural formula of BFA is depicted

In initial testing, the crude extract from cultured broth of fungal strain *Penicillium* sp. FKI-7127 showed pronounced inhibition on DENV growth as determined from the reduction of antigen level in infectious culture fluid (data not shown). After optimization of culture condition which gave the highest inhibition or highest yield, two rounds of fractionation from large-scale culture broth were performed. Thirty-six final fractions were collected from HPLC fractionation, and each fraction was tested for its antiviral activity. We found that fraction #17 that showed the highest peak in chromatogram (Fig. 1a) is the active fraction.

Inhibitory effect and cytotoxicity of fraction #17 were further evaluated in increasing dilution (Fig. 1b). Fraction #17 inhibited DENV-2 in a dose-dependent manner. The 50% cytotoxic concentration (CC_50_) of the fraction after 5 days of simultaneous incubation was > 16×, the lowest dilution tested.

We next performed the identification of active components in fraction #17. Using HR-ESI-MS and NMR methods, brefeldin A was revealed as an active compound (Fig. 1c).

**Fig. 2:**
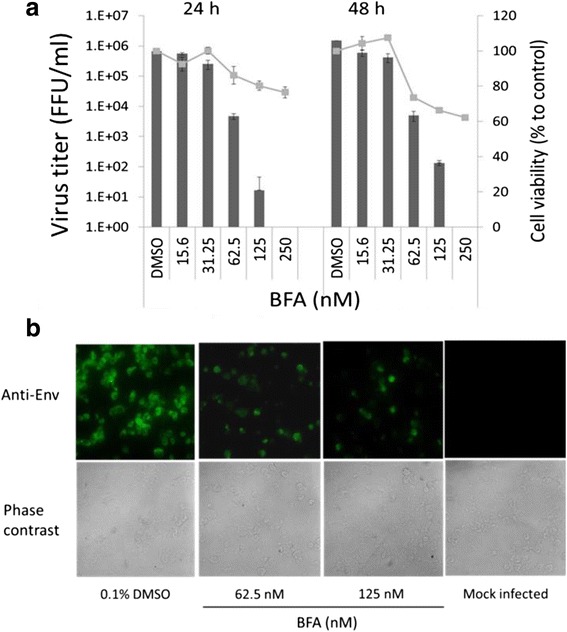
Identification of anti-DENV activity of brefeldin A by virus titration and immunofluorescence assay or IFA. **a** Vero cells in 96-well plates were infected with DENV at an MOI of 0.5 in the presence of compound at indicated concentrations or 0.1% DMSO. After 24 h or 48 h incubation, ICF was harvested and virus titer was determined by focus assay as described in the “Materials and methods” section. In separated experiments, Vero cells in 96-well plates were treated with the compounds at different concentrations without virus infection. After 24 h or 48 h incubation, cell viability was determined by MTT assay as described in the “Materials and methods” section. **b** Vero cells in 24-well plates were infected with DENV at an MOI of 0.5 and added with the compounds at indicated concentrations. After 48 h incubation, the cells were recovered, spotted onto glass slides, and stained with 12D11 monoclonal antibody which react to viral envelope protein

To confirm antiviral activity of BFA, we purchased BFA (Wako, Japan) and performed virus titration and immunofluorescence assay (IFA). BFA effectively inhibited DENV-2 growth as determined by virus titer. At a concentration of 250 nM, no virus was detected both at 24 and 48 h postinfection (Fig. 2a). Half maximal inhibitory concentration (IC_50_) value and CC_50_ of BFA at 48 h postinfection were 54.6 ± 0.9 and 2000 nM, respectively (Table 1). In line with virus titration results, IFA staining showed significantly decreased viral protein level by addition of BFA at concentrations of 62.5 and 125 nM (as indicated by green fluorescence) suggesting that DENV-2 replication was strongly inhibited (Fig. 2b). Taken together, these results demonstrated BFA isolated from fungal strain *Penicillium* sp. FKI-7127 as an inhibitory agent against DENV.

**Table 1:** Antiviral activity of BFA against DENV-1 to 4, ZIKV, and JEV in Vero cell

IC_50_ (nM)^a^						CC_50_ (nM)^b^
DENV-1	DENV-2	DENV-3	DENV-4	ZIKV	JEV	
61.3 ± 13.5	54.6 ± 0.9	57.9 ± 0.1	65.7 ± 6.3	54.8 ± 0.4	58.4 ± 0.3	2000

All calculation was performed by using GraphPad Prism software. All values are the results from two independent experiments

^a^IC_50_—50% inhibitory concentration of BFA were calculated from the results of the virus titer determined by focus-forming assay

^b^CC_50_—50% cytotoxic concentration of BFA were calculated from the dose-response curve

BFA is a fungal secondary metabolite which was first isolated from *Penicillium decumbens* [12]. BFA has various biological actions including antitumor and antibacterial activities [13]. BFA has also been reported to have antiviral activities against some viruses including poliovirus [14] and Rotavirus [15]. Recently, Zhou et al. demonstrated the inhibitory effect of BFA on Japanese encephalitis virus (JEV) in BHK-21 cells [16]. To our knowledge, this is the first report to demonstrate antiviral activity of BFA on DENV.

**Fig. 3:**
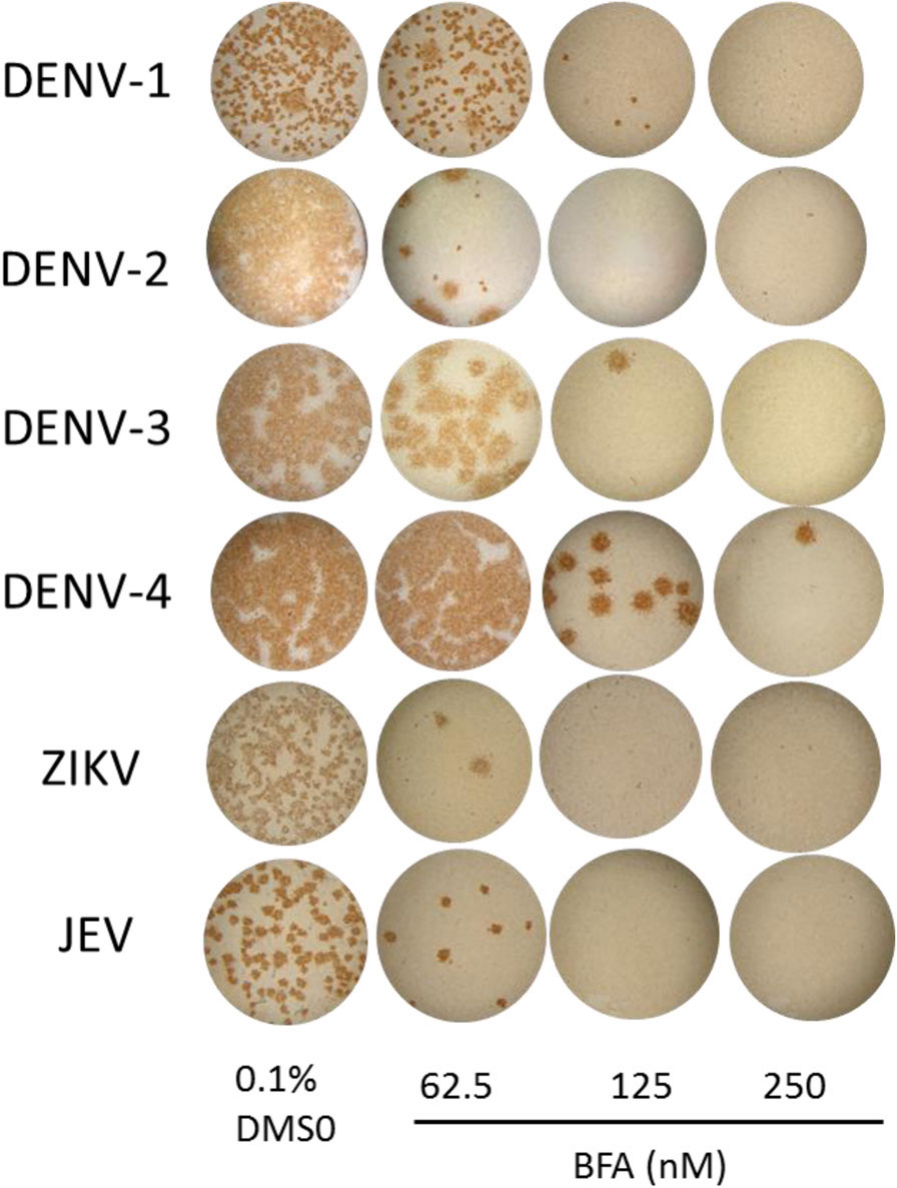
Inhibitory effect of BFA on all DENV serotypes, ZIKV, and JEV. Vero cells in 96-well plates were infected with DENV-1, 2, 3, and 4, ZIKV, or JEV in the presence of BFA at indicated concentration or 0.1% DMSO. Forty-eight hours after infection/treatment, ICFs were harvested and then infected into fresh cells for focus reduction assay as described in the “Materials and methods” section. The image shown is a representative result from two experiments

Antiviral activity of BFA against all DENV serotypes as well as two related viruses including ZIKV and JEV was also analyzed by focus reduction assay. Addition of BFA reduced focus number in a dose-dependent manner which indicates virus inhibition. BFA inhibited not only DENV-2 but also all other serotypes. Furthermore, strong inhibition of ZIKV and JEV by BFA was also demonstrated (Fig. 3). Determined by virus titer, the EC_50_ of BFA against DENV-1, 3, and 4, ZIKV, and JEV at 48 h postinfection/treatment were 61.32 ± 13.5, 57.9 ± 0.1, 65.7 ± 6.3, 54.8 ± 0.4, and 58.4 ± 0.3 nM, respectively (Table 1).

**Fig. 4:**
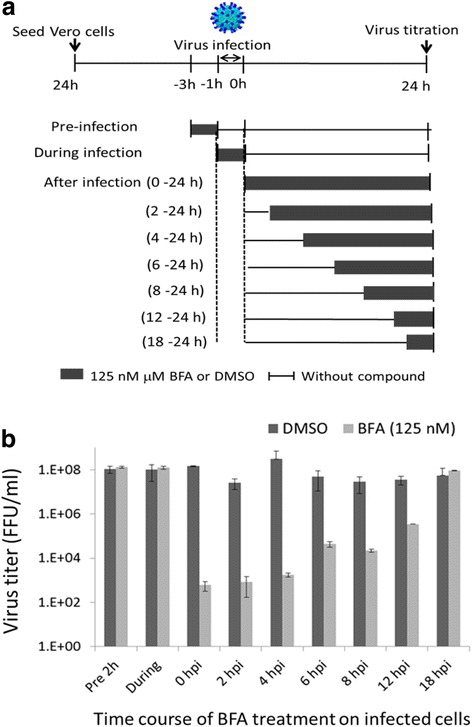
Time-of-addition studies. **a** Schematic illustration of time-of-addition studies for treatment with BFA. Vero cells in 96-well plates were treated with 125 nM BFA at different time points before, during, and after DENV infection (MOI of 5). For after-infection assay, BFA was added at seven different time points after virus exposure. **b** After 24 h postinfection, released virus titer was determined by focus assay as described in the “Materials and methods” section. Error bars represent standard errors of two independent experiments

In order to identify the window in the DENV replication cycle when BFA exerts its antiviral effect, time-of-addition studies were performed in different treatments (Fig. 4a). During a single flavivirus life cycle, viral proteins are translated from genomic RNA in the first 1–5 h postinfection (hpi) followed by viral RNA synthesis which occurs after 5 hpi and progeny virus assembly and release after 12 hpi [17]. As shown in Fig. 4b, BFA does not interfere in the DENV entry process in the host cells. A significant reduction of the DENV titer was observed when BFA was added at the 0-hpi up to 4-hpi time points. After 4 hpi, inhibitory effect of BFA was gradually reduced. Addition of BFA at 18 hpi resulted in complete loss of inhibition of DENV replication. These results suggested that BFA inhibits DENV at an early phase in the viral replication cycle that occurs after viral entry.

The time-of-addition study results are in line with an already known mode of action of BFA. BFA has been known to inhibit protein transport from the endoplasmic reticulum (ER) to the Golgi apparatus indirectly by interfering with the function of the Golgi apparatus [18]. BFA disturbs maturation and egress of herpes simplex virus particles during infection [19]. BFA has also been reported to interfere processing and secretion of the envelope of glycoproteins of HIV-1 in T-lymphoblast cells leading to inhibition of viral particle formation [20]. In case of poliovirus, BFA inhibits viral RNA synthesis by preventing the formation of secretory vesicles [21]. We speculate that, like other enveloped viruses, BFA inhibits the maturation of the DENV, ZIKV, and JEV by directly blocking the trafficking of glycoprotein from the ER to Golgi apparatus leading to the prevention of formation and release of the viruses from infected cells. In addition in our study, no inhibitory effect of BFA on DENV was shown in C6/36 cells (data not shown) indicating that BFA could not block intracellular protein transport in mosquito cell line.

Despite the fact that BFA possesses antiviral activity as demonstrated in this study and other previous studies, its toxicity would become a crucial issue in order to develop it as antiviral agents. Toxicity of BFA indeed is not unexpected because it targets Golgi apparatus which eventually causes cell death. However, BFA could be served as a lead compound. In the future, it can be structurally and phenotypically optimized by reducing its toxicity. Furthermore, its antiviral activity can also be improved through derivative analyses or another approaches.

Aside from its antiviral property, mechanism of disruption of the proper vesicular transport between ER and Golgi by BFA which is a critical step for the viral replication and release could provide a new tool to characterize the lifecycle of the virus. It is also possible to help researchers develop novel inhibitors of DENV and other viruses.

## Conclusion

In conclusion, here we isolated, identified, and characterized an anti-DENV agent of fungus-derived BFA which is potentially used as a lead compound for drug development of anti-DENV and other related viruses. Fungal secondary metabolites are a potential and valuable source in drug screening for the development of antiviral agents.

## Abbreviations

BFA: Brefeldin A
CC_50_: The 50% cytotoxic concentration
DENV: Dengue virus
DF: Dengue fever
DHF: Dengue hemorrhagic fever
DMSO: Dimethyl sulfoxide
ELISA: Enzyme-linked immunosorbent assay
HPLC: High-performance liquid chromatography
IC_50_: Half maximal inhibitory concentration
IFA: Immunofluorescence assay
JEV: Japanese encephalitis virus
ZIKV: Zika virus

## References

[1] Simmons CP, Farrar JJ, Nguyen VV, Wills B (2012). Dengue. N Engl J Med.

[2] Bhatt S, Gething PW, Brady OJ, Messina JP, Farlow AW, Moyes CL (2013). The global distribution and burden of dengue. Nature.

[3] Cragg GM, Newman DJ (2013). Natural products: a continuing source of novel drug leads. Biochim Biophys Acta.

[4] Demain AL, Martens E (2017). Production of valuable compounds by molds and yeasts. J Antibiot.

[5] Strobel G, Yang X, Sears J, Kramer R, Sidhu RS, Hess WM (1996). Taxol from *Pestalotiopsis microspora*, an endophytic fungus of *Taxus wallachiana*. Microbiology.

[6] Radić N, Strukelj B (2012). Endophytic fungi: the treasure chest of antibacterial substances. Phytomedicine.

[7] Bunyapaiboonsri T, Yoiprommarat S, Srikitikulchai P, Srichomthong K, Lumyong S (2010). Oblongolides from the endophytic fungus *Phomopsis* sp. BCC 9789. J Nat Prod.

[8] Roy BG (2017). Potential of small-molecule fungal metabolites in antiviral chemotherapy. Antiviral Chem Chemother.

[9] Estoppey D, Lee CM, Janoschke M, Lee BH, Wan KF, Dong H (2017). The natural product cavinafungin selectively interferes with Zika and dengue virus replication by inhibition of the host signal peptidase. Cell Rep.

[10] Ngwe Tun MM, Kyaw AK, Makki N, Muthugala R, Nabeshima T, Inoue S (2016). Characterization of the 2013 dengue epidemic in Myanmar with dengue virus 1 as the dominant serotype. Infect Genet Evol.

[11] Raekiansyah M, Espada-Murao LA, Okamoto K, Kubo T, Morita K (2014). Dengue virus neither directly mediates hyperpermeability nor enhances tumor necrosis factor-α-induced permeability in vitro. Jpn J Infect Dis.

[12] Singleton VL, Bohonos N, Ullstrup AJ (1958). Decumbin, a new compound from a species of *PenicilIium*. Nature.

[13] Betina V (1992). Biological effects of the antibiotic brefeldin A (decumbin, cyanein, ascotoxin, synergisidin): a retrospective. Folia Microbiol (Praha).

[14] Cuconati A, Molla A, Wimmer E, Brefeldin A (1998). Inhibits cell-free, de novo synthesis of poliovirus. J Virol.

[15] Mirazimi A, von Bonsdorff CH, Svensson L (1996). Effect of brefeldin A on rotavirus assembly and oligosaccharide processing. Virology.

[16] Zhou J, Wang SQ, Wei JC, Zhang XM, Gao ZC, Liu K (2017). Mx is not responsible for the antiviral activity of interferon-α against Japanese encephalitis virus. Viruses.

[17] Chambers TJ, Hahn CS, Galler R, Rice CM (1990). Flavivirus genome organization, expression, and replication. Annu Rev Microbiol.

[18] Lippincott-Schwartz J, Yuan LC, Bonifacino JS, Klausner RD (1989). Rapid redistribution of Golgi proteins into the ER in cells treated with brefeldin A: evidence for membrane cycling from Golgi to ER. Cell.

[19] Cheung P, Banfield BW, Tufaro F, Brefeldin A (1991). Arrests the maturation and egress of herpes simplex virus particles during infection. J Virol.

[20] Pal R, Mumbauer S, Hoke GM, Takahashi A, Sarngadharan MG, Brefeldin A (1991). Inhibits the processing and secretion of envelope glycoproteins of human immunodeficiency virus type 1. AIDS Res Hum Retrovir.

[21] Maynell LA, Kirkegaard K, Klymkowsky MW (1992). Inhibition of poliovirus RNA synthesis by brefeldin A. J Virol.

